# Radiation-induced meningeal osteosarcoma of tentorium cerebelli with intradural spinal metastases

**DOI:** 10.4103/2152-7806.63909

**Published:** 2010-05-31

**Authors:** John E. Ziewacz, Jae W. Song, Mila Blaivas, Lynda J.S. Yang

**Affiliations:** Department of Neurosurgery, University of Michigan Health System, Ann Arbor, MI, USA; 1 Department of Pathology, University of Michigan Health System, Ann Arbor, MI, USA

**Keywords:** Meninges osteosarcoma, Radiation therapy, Spinal metastasis, Tentorium cerebelli

## Abstract

**Background:**

Primary meningeal osteosarcomas and radiation-induced extraosseous tumors are extremely rare. We encountered a patient with a radiation-induced meningeal osteosarcoma with metastatic spread.

**Case Description:**

A 54-year-old man presented with a 2-week history of nausea, vomiting, and ataxia. CT and MRI studies revealed an extra-axial, dural-based mass in the posterior fossa arising from the tentorium cerebelli. The patient underwent complete resection of the tumor with adjuvant chemotherapy. Histopathologic analysis revealed chondroblastic osteosarcoma. Tumor recurrence was observed 9 months after initial diagnosis, and adjuvant radiation therapy was administered. The intracranial disease stabilized; however, multiple cervico-thoracic spinal metastases were discovered 15 months after initial diagnosis. The patient expired 16 months after initial diagnosis.

**Conclusion:**

Meningeal osteosarcomas are rare lesions that can metastasize and should be considered in the differential diagnosis for dural-based lesions, especially in the case of previous radiation therapy.

## INTRODUCTION

Extraskeletal osteosarcomas account for 4% of all osteosarcomas and are rarely found in the central nervous system (CNS).[4] Primary CNS osteosarcomas may arise from brain parenchyma[3,6,9,14,20,23,25,29] or from meninges,[1,5,7,10,17,21,26–28,31] with the latter being exceedingly rare.[27] Additionally, nonosseous radiation-induced osteosarcomas are rare.[13] Only one previous case of a radiation-induced meningeal osteosarcoma has been reported.[7] We present an additional case of a radiation-induced meningeal osteosarcoma, and the first with documented metastatic spread.

## CASE DESCRIPTION

### History

**Figure 1:**
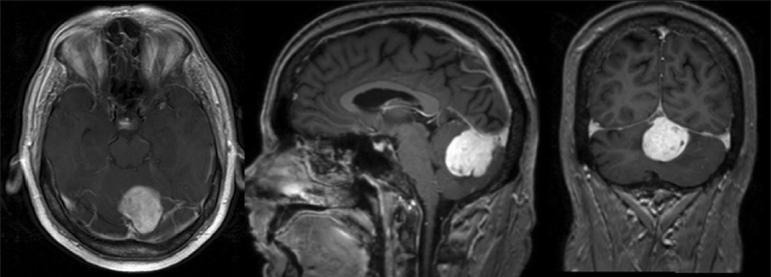
Axial (*left*), sagittal (*center*), and coronal (*right*) T1-weighted MRIs with gadolinium enhancement illustrating suboccipital mass at patient's initial presentation

A 54-year-old man with a remote history of stage III Hodgkin's lymphoma treated 24 years earlier by mantle irradiation presented to our institution with a 2-week history of nausea, vomiting, and ataxia. On examination, he exhibited mild dysmetria and difficulty with rapid alternating movements in the left upper extremity, and a mildly wide-based gait. Brain magnetic resonance imaging (MRI) revealed a 3.6 cm × 3.4 cm × 3.2 cm homogenously enhancing extra-axial mass arising from the tentorium cerebelli, eccentric to the left and crossing the midline [Figure 1]. Bony involvement was not evident. There was surrounding edema and mild mass-effect on the fourth ventricle, but no evidence of obstructive hydrocephalus. The presumptive preoperative diagnosis was meningioma, given the homogenous enhancement, extra-axial location, and association with the tentorium. Computed tomography (CT) of the chest, abdomen, and pelvis revealed no evidence of metastatic disease. 

### Operation

**Figure 2:**
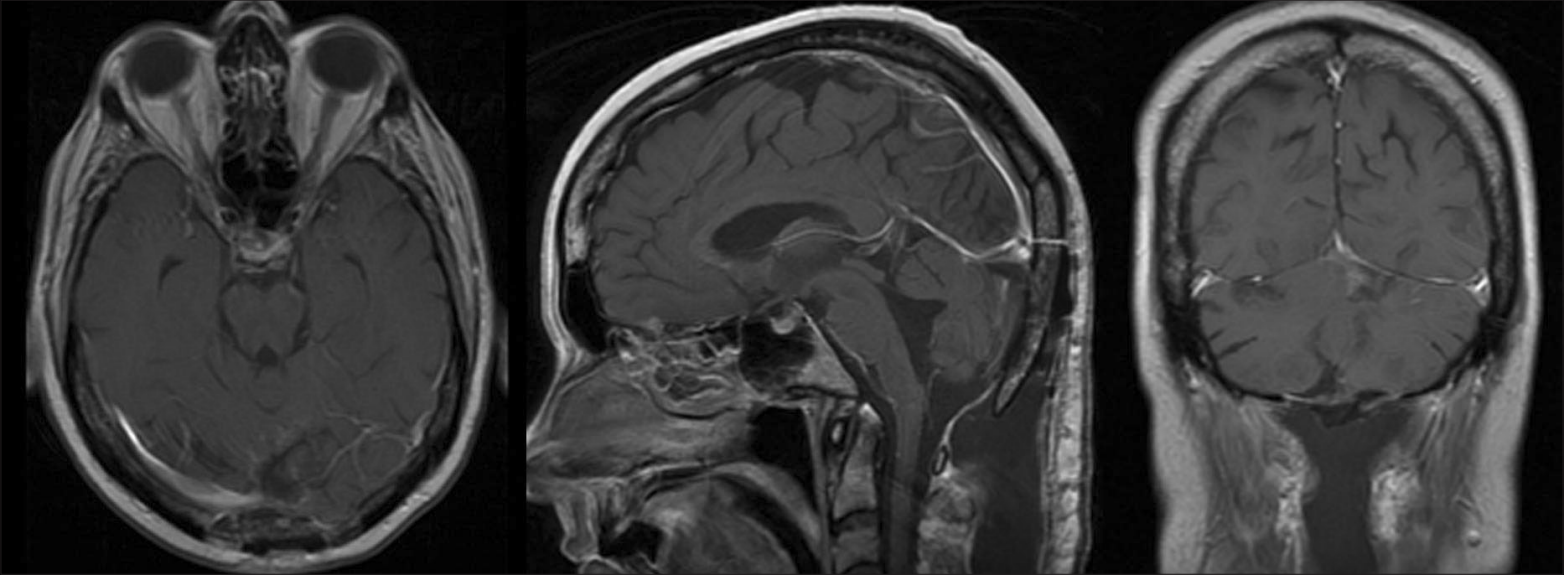
Axial (*left*), sagittal (*center*), and coronal (*right*) T1-weighted MRIs with gadolinium enhancement illustrating extent of resection

The patient underwent suboccipital craniotomy for resection of the mass with placement of a ventricular catheter. The procedure was uncomplicated, and the patient was discharged after a routine postoperative course.Although he continued to experience some mild left upper extremity dysmetria, his nausea, vomiting, and ataxia improved. Postoperative MRI revealed no obvious evidence of residual disease [Figure 2].

### Histological findings

**Figure 3:**
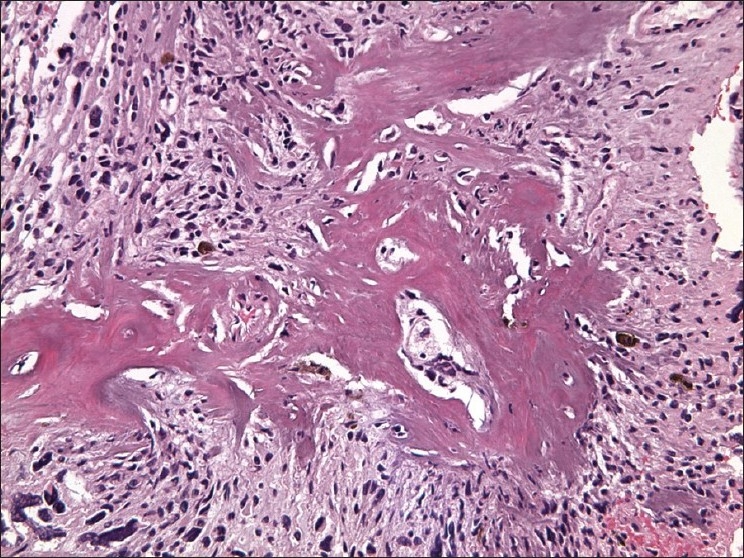
Histology section of resected tumor reveals malignant osteoid formation (hematoxylin and eosin staining; ×200 magnification)

Histopathologic analysis of the mass revealed a chondroblastic osteosarcoma with highly malignant features, including bizarre neoplastic cells, abnormal mitotic figures, and a MIB-1 index of 90%. Formalin-fixed paraffin-embedded sections of the tumor were stained with hematoxylin and eosin (HandE), trichrome, as well as several immunohistochemical markers. The mitotically active neoplasm was composed of pleomorphic cells with hyperchromatic multinucleated forms and focal osteoid formation [Figure 3]. Neoplastic cells were positive for vimentin, but negative for epithelial membrane antigen, thereby excluding the diagnosis of a meningioma. Staining for CD68 was also negative. Regions of cartilage formation were positive for S-100 protein. Infiltration of adjacent brain parenchyma by neoplastic cells was apparent in sections stained for neurofilament protein and synaptophysin. These features were thought to be consistent with a radiation-induced chondroblastic osteosarcoma.

Notably, the patient′s outside hospital records indicated that the 20 Gy radiation dose he received for Hodgkin′s lymphoma 20 years prior had been performed with the neck extended, and the field treated included the location of his posterior fossa tumor.

### Postoperative treatment

**Figure 4:**
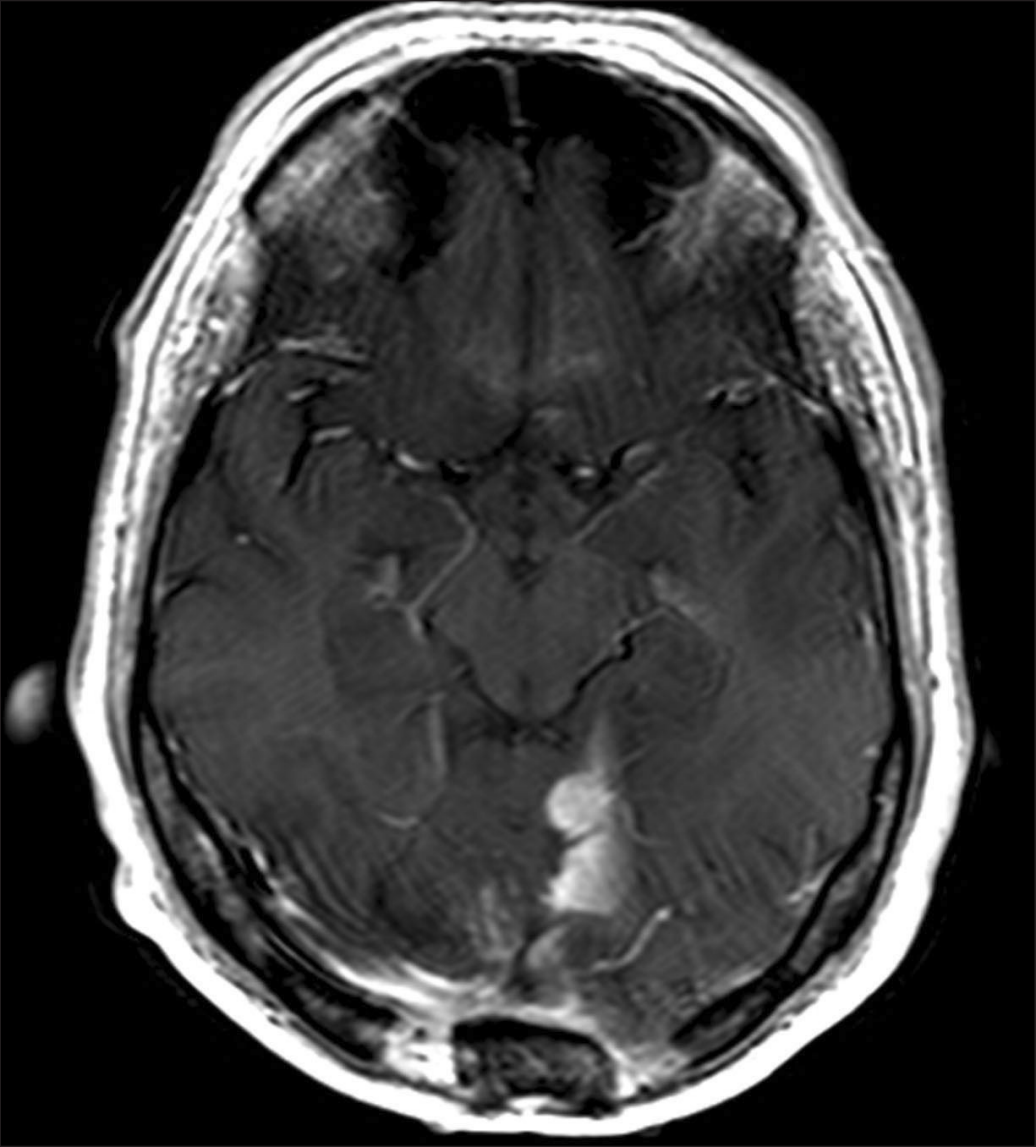
Axial T1-weighted MRI with gadolinium enhancement showing recurrence of lesion

**Figure 5:**
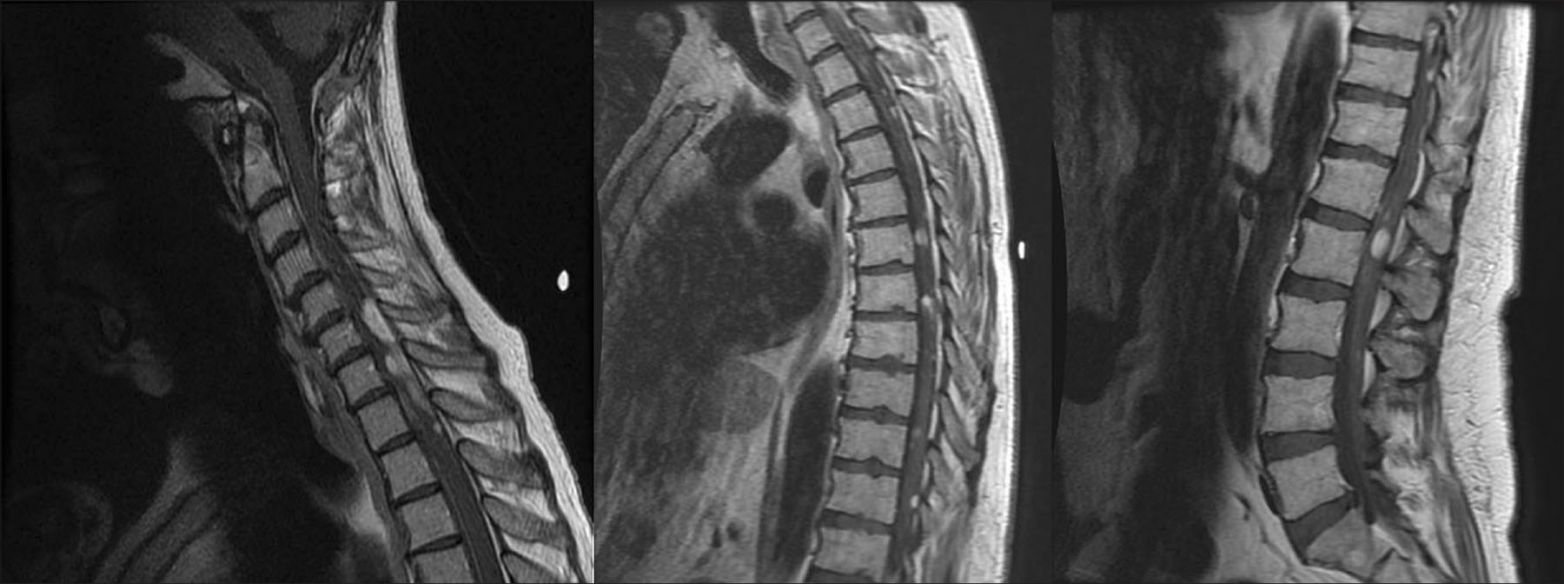
Sagittal T1-weighted MRIs with gadolinium enhancement showing multiple intradural, intra-, and extramedullary metastases

On the basis of the pathology findings, the patient underwent two rounds of ifosfamide and doxorubicin chemotherapy. Radiation therapy was initially deferred due to both previous irradiation to the area and relative radioresistant nature of these tumors.[12,15] Each round of chemotherapy was associated with intractable nausea and vomiting requiring hospitalization. The second hospitalization was complicated by a deep vein thrombosis requiring anticoagulation, organizing pneumonia with bronchiolitis obliterans, and methicillin-resistant *Staphylococcus aureus* bacteremia. The patient recovered from these hospitalizations, and he was at his neurologic baseline. Nine months after his initial presentation, routine MRI revealed local recurrence of the disease [Figure 4]. Radiation therapy was scheduled. However, when the patient presented for therapy, he was experiencing significant ataxia, nausea, and vomiting. A noncontrast head CT was performed, which showed the known recurrence as well as obstructive hydrocephalus. The patient and his family declined further resection but wished to proceed with palliative ventriculoperitoneal shunting. This procedure was performed without complications. After shunt placement, the patient underwent intensity-modulated radiation therapy with a total dose of 65 Gy to the tumor bed. The patient′s symptoms improved and follow-up imaging revealed a reduction in tumor size. He remained stable for 3 months after the completion of radiation therapy, but then experienced progressively worsening bilateral lower extremity weakness over the course of 3 days. MRI of the thoracolumbar spine performed as part of a work-up for his weakness revealed multiple intradural, intra-, and extramedullary masses, as well as diffuse epidural enhancement consistent with metastatic disease [Figure 5]. Palliative radiation was performed for symptom control. The patient was discharged to hospice where he succumbed to his disease 16 months after initial presentation.

## DISCUSSION

Primary osteosarcomas typically occur in the metaphyses of the long bones and rarely present in extraskeletal locations.[4] Also, radiation exposure has long been known to be associated with the development of osteosarcoma.[2] In one review of more than 1,200 patients with osteosarcoma, 5.5% were thought to be directly related to therapeutic or incidental irradiation, with only seven occurring in extraskeletal locations.[13] Interestingly, 9 of 66 patients who developed radiation-induced osteosarcomas underwent radiation for treatment of Hodgkin′s lymphoma.[13] On the basis of the definition by Cahan *et al*.[8] an osteosarcoma can be considered radiation-induced if it meets the following criteria: (1) It possesses microscopic or X-ray evidence of nonmalignancy; (2) radiation therapy administered to the patient results in tumor developing within the path of the radiation beam; (3) patient experiences a relatively long asymptomatic period; and (4) the diagnosis of osteosarcoma is confirmed by histopathology. To the best of our knowledge, only the present case and one other reported case of a meningeal osteosarcoma fit these criteria. In our case, the latent period from radiation exposure to diagnosis was 24 years, which is similar to the 22 years between radiation exposure and diagnosis in the other reported meningeal case[7] and is within the observed range for the interval between radiation exposure and diagnosis of radiation-induced osteosarcoma.[13]

Meningeal osteosarcomas are exceedingly rare tumors reported infrequently in both humans and animals.[1,5,7,10,17,21,22,24,26–28,31] They are thought to arise from malignant conversion of multipotential mesenchymal cells that give rise to the meninges, blood vessels, and membranous cranium, thus giving rise to sarcomatous tumors of various differentiations.[16,19,27] They have not been previously known to metastasize.[27]

Diagnosis of meningeal osteosarcoma is based initially on CT and MRI findings, although these findings are not pathognomonic and are often first presumed to be meningiomas, given their dural base, extra-axial location, and enhancement pattern.[27,30] Cerebral angiography has been performed in some cases, and the lesions were found to be space-occupying lesions with blood supply mostly from meningeal vessels from the external and internal carotid arteries.[27,31] Given the difficulty in distinguishing these masses from meningiomas radiographically, definitive diagnosis relies on histopathologic confirmation. To our knowledge, metabolic imaging studies such as Positron Emission Tomography (PET) or MR spectroscopy have not been used for differentiation.

It is difficult to determine the appropriate therapy for meningeal osteosarcoma as there have been so few cases reported. Given the paucity of cases, most intracranial osteosarcomas, including meningeal osteosarcomas, are treated according to therapies that have demonstrated efficacy for osteosarcomas elsewhere.[7,11,18,26] This typically includes surgical resection with adjuvant chemotherapy, which was associated with a progression-free survival of 2 years in another reported case of radiation-induced meningeal osteosarcoma.[7] Other regimens, including surgery alone and surgery with adjuvant radiation, have also shown promise with progression-free survival at 2- and 3-year follow-up, respectively.[27,28] Despite these encouraging results, historically the prognosis for intracranial osteosarcomas, including meningiomas, has been considered poor, and the longest follow-up to date for a meningeal osteosarcoma has been 33 months.[27] The present study highlights the aggressive course that these tumors can take despite aggressive resection, adjuvant chemotherapy, and radiation therapy. Additionally, our case represents the first known report of a primary meningeal osteosarcoma with metastatic spread.

## CONCLUSION

Primary meningeal osteosarcomas are exceedingly rare tumors, as are extraosseous radiation-induced osteosarcomas. Our case of a radiation-induced primary meningeal osteosarcoma is the first to document metastatic spread. The often aggressive nature of this disease, despite maximal therapy, and the potential for metastatic spread, is also highlighted by this study. Thus, it is important to include osteosarcoma in the differential diagnosis for patients with dural-based masses, especially those with a history of previous radiation therapy.
